# Effect of Heat Treatments on the Microstructure and Properties of 18Ni300 Maraging Steel Produced by Selective Laser Melting

**DOI:** 10.3390/ma18102284

**Published:** 2025-05-14

**Authors:** Jun Hu, Lei Zhang, Xuanzheng Wang, Wenzhao Lin, Pingang Wei, Yiwei Cao, Juanqi Zhang, Kai Sun, Bing Yang, Wentao Li

**Affiliations:** 1 CNOOC Safety &Technology Service Co., Ltd., Tianjin 300450, China; bosun_hj@126.com (J.H.); wxz0209@163.com (X.W.); siriuslwz@163.com (W.L.); 16811@163.com (P.W.); 18020063338@163.com (Y.C.); zjqoffcor@foxmail.com (J.Z.); sunkai8905@126.com (K.S.); lwtsd@126.com (W.L.); 2 School of Power & Mechanical Engineering, Wuhan University, Wuhan 430072, China

**Keywords:** SLM, maraging steel, tensile ductility, aging treatment, solution treatment

## Abstract

The microstructure and tensile properties of 18Ni-300 maraging steel manufactured by selective laser melting (SLM) were investigated after different heat treatments and compared to the original samples. Heat treatment alters the microscopic morphology of the original sample, and the differences in the cross-sectional and longitudinal sectional morphology of the original sample become indistinguishable after heat treatment. Cellular and long strip structures can be observed in the original and aged samples. After solution aging, the cellular and long strip structures completely disappeared, being transformed into parallel and almost equal-length plate martensite. Additionally, inverted austenite and Ni_3_(Ti, Al, Mo) precipitates were present. The microhardness increased from 310 HV to 710 HV, the nanohardness rose to 7.7 GPa, tensile strength reached 2068 MPa, and elongation to fracture improved to 4.5%. These optimal properties were achieved with solution treatment at 820 °C for 2 h and aging at 490 °C for 7 h.

## 1. Introduction

Selective laser melting (SLM) is one of the most widely used metal additive manufacturing technologies in the industry, offering significant advantages in the rapid prototyping of complex structures and the production of items with high surface dimensional accuracy and low surface roughness (Ra = 30–50 μm). Maraging steel, containing 18% nickel, is an ideal candidate for additive manufacturing processes due to its low carbon content and strong resistance to cracking during the rapid cooling associated with additive manufacturing [1,2]. Additionally, the rapid solidification during additive manufacturing promotes the phase transition from austenite to martensite and forms fine honeycomb grains, thereby enhancing the strength of the steel [3,4,5].

To effectively apply the SLM process for fabricating key components of 18Ni300, a comprehensive study on the relationship between the process parameters, microstructure, and mechanical properties is essential. Relevant experimental studies have been conducted within the academic community. Currently, most research on 18% nickel maraging steels focuses on process optimization, microstructure characterization, mechanical property evaluation, and the exploration of heat treatment processes for specific additive manufacturing technologies. For instance, Mutua et al. investigated the effects of various process parameters on the densification behavior, surface morphology, microstructure, and mechanical properties of 18Ni300 maraging steel produced by SLM [6]. Additionally, Suzuki et al. systematically examined how laser power and scanning speed affect the relative density, melt pool depth, and Vickers hardness of selectively laser-melted maraging steels. Their results indicated significant variations in relative density, melt pool depth, and Vickers hardness, even when the volumetric energy density values were the same [7].

To fully realize the performance potential of 18Ni300 maraging steel, the selection and implementation of appropriate heat treatment processes are crucial. The influence of various heat treatment processes on its properties has been extensively explored in the academic community. For instance, Bai et al. used SLM technology to manufacture high-performance 300-grade maraging steel and found that the hardness and tensile strength of the SLM specimens, after aging treatment, met the standard forging requirements and were comparable to those of standard martensitic aging steel, according to the United States Iron and Steel Industry Association [4]. To enhance the quality of the molds, Bai et al. conducted solution treatment and solution aging treatment separately. Their results showed that solution treatment improved the mechanical finishing of the mold and increased surface accuracy [8]. Yin et al. investigated the effects of aging temperature and aging time on the microstructure, mechanical properties (hardness, strength, and ductility), and tribological properties of SLM 18Ni300 maraging steel [9]. Xu et al. examined the microstructural changes, phase transitions, and mechanical properties of arc additive manufacturing 18Ni300, finding that aging conditions significantly impacted the strength and wear resistance of SLM martensitic aging steels. Specifically, aging at 490 °C for 3 h yielded the maximum strength and optimal wear resistance [10]. Additionally, a review article compared the mechanical properties of 18Ni300 maraging steel prepared by SLM and conventional forging after heat treatment [11]. SLM technology enables the design of internal flow channels, complex cooling channels, and conformal cooling flow paths, providing excellent dynamic balance performance and effectively improving the deformation rate of mold products. Thus, the preparation of 18Ni300 molds using SLM technology holds significant development potential.

Therefore, this paper will investigate the influence mechanisms of two different heat treatment processes—aging and solution treatment—as well as the effects of varying aging temperatures and durations on the microstructure and mechanical properties of maraging steel. This research will provide both theoretical and experimental foundations for understanding the evolution of its microstructure, optimizing the manufacturing process, enhancing material properties, and supporting its application in aerospace, automotive manufacturing, precision molds, and other fields.

## 2. Materials and Methods

### 2.1. Materials

**Figure 1:**
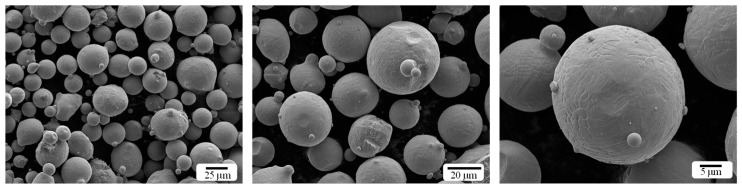
Powder morphology of 18-Ni300 alloy.

The morphology and main chemical composition of maraging steel powder (MS) are shown in Figure 1 and Table 1, respectively. The median diameter (D50) was measured as 15–53 µm using laser diffraction. An SLM device (EP-M300D, Eplus 3D, Hangzhou, China) was used to fabricate the experimental parts. The optimized process parameters are as follows: laser power of 300 W, hatch spacing of 110 µm, scanning rate of 920 mm/s, layer thickness of 50 µm, and spot diameter of 76 µm.

After fabrication, the specimens were heated using an XMT-80 (Shanghai YATAI instrument Co., Ltd., Shanghai, China) muffle furnace, with water quenching as the cooling method. Based on the heating rate of the furnace and the phase change points of the maraging steel [12,13,14,15,16], the solution temperature was set to 820 °C, and the aging temperatures were set to 490 °C and 700 °C, with heat preservation (HP) for 4–10 h. The heat treatment parameters are shown in Table 2.

### 2.2. Methods

Phase structure, orientation, and phase mass fraction were measured using an X-ray diffractometer (TDM-10, Dandong Tongda Science & Technology Co., Ltd., Dandong, China). Cu Kα radiation was used as the X-ray source (λ = 0.15418 nm), with a scanning rate of 1°/min and a scanning range of 10–90°. A light microscope (Carl Zeiss Microscopy GmbH, Jena, Germany) was employed to observe the polished samples after acid etching with nitric acid (Optical Microscope, OM). High-magnification morphological analysis and electron back-scattered diffraction (EBSD) were conducted using a field emission scanning electron microscope (MIRA 3) equipped with an X-Max 20 spectrometer (Aztec Energy, Wauchula, FL, USA). The test conditions for SEM topography observation were working distance of 5–17 mm and accelerating voltage of 5–20 kV. TEM topography was observed using a transmission electron microscope (FEI Talos F200X G2, Shanghai, China).

**Figure 2:**
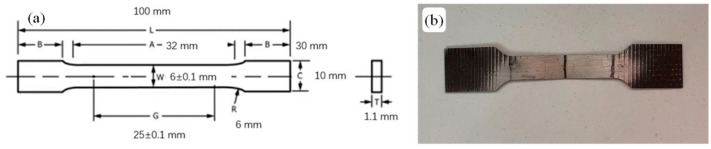
Tensile samples: (**a**) size diagram and (**b**) real sample.

According to ASTM E8 standards [17], the tensile specimen size is shown in Figure 2 and was tested on an MTS Landmark 370.02 mechanical testing machine (Beijing, China) with a displacement rate of 1.5 mm/min.

A micro-Vickers hardness tester (Micro Vicker HV-1000A, Shandong, China) was used to measure the microhardness of the specimens after polishing. The test was conducted with a load of 500 gf and a dwell time of 10 s, with the average value calculated from six repeated tests. A nanoindentation instrument (NANO G200, Shanghai, China) was used to measure the nanohardness and elastic modulus of the specimens. Six measurements were taken for each sample, and the average value was computed. The Oliver–Pharr method was employed to determine the hardness and modulus of elasticity of the specimens.

## 3. Results and Discussion

### 3.1. Microstructures

**Figure 3:**
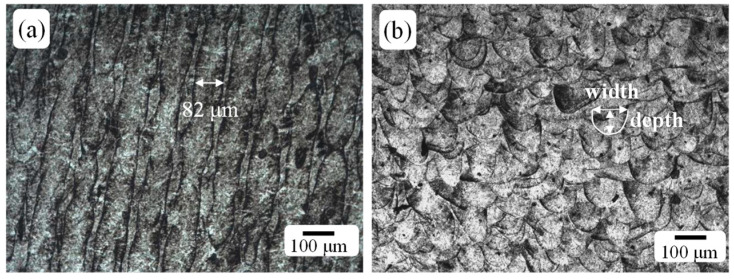
OM images of horizontal and vertical surface of AM specimen: (**a**) horizontal surface and (**b**) vertical surface.

The optical microscope (OM) microstructure of the horizontal and vertical surfaces of the additive manufacturing (AM) samples is presented in Figure 3. The AM specimen clearly displays the typical microstructure of steels manufactured by SLM. The horizontal surface structure is primarily columnar, with a width of approximately 82 μm, which is consistent with the laser spot size used in the SLM process. On the vertical surface, the fish scale-like morphology of the melt pool is evident. The width of the top of the melt pool ranges from 90 to 110 μm, and the depth of the melt pool is about 96 μm, which is generally consistent with the powder layer thickness.

**Figure 4:**
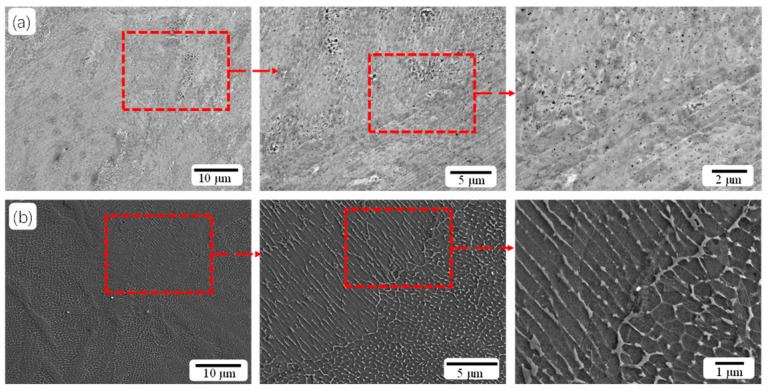
SEM images of the samples after heat treatment: (**a**) AM and (**b**) AT 490-7.

Figure 4 shows SEM images of the AM and AT 490-7 samples. The AM specimen clearly exhibits cellular and elongated structures. After aging treatment, the microstructure of the sample (AT 490-7) does not change significantly; cellular and elongated structures remain dominant. However, after aging, the cell-like and elongated structures appear fractured at their borders, and the distribution of white phases at the boundaries is not very uniform, indicating possible element enrichment and segregation. High-magnification SEM observations reveal distinct details in the cellular structure of the AM sample, with the diameter of the equivalent circle measured to be approximately 0.4 μm. This notable feature is primarily due to the extremely fast cooling rate during the SLM process, which significantly promotes the refinement of tissue grains [18,19].

**Figure 5:**
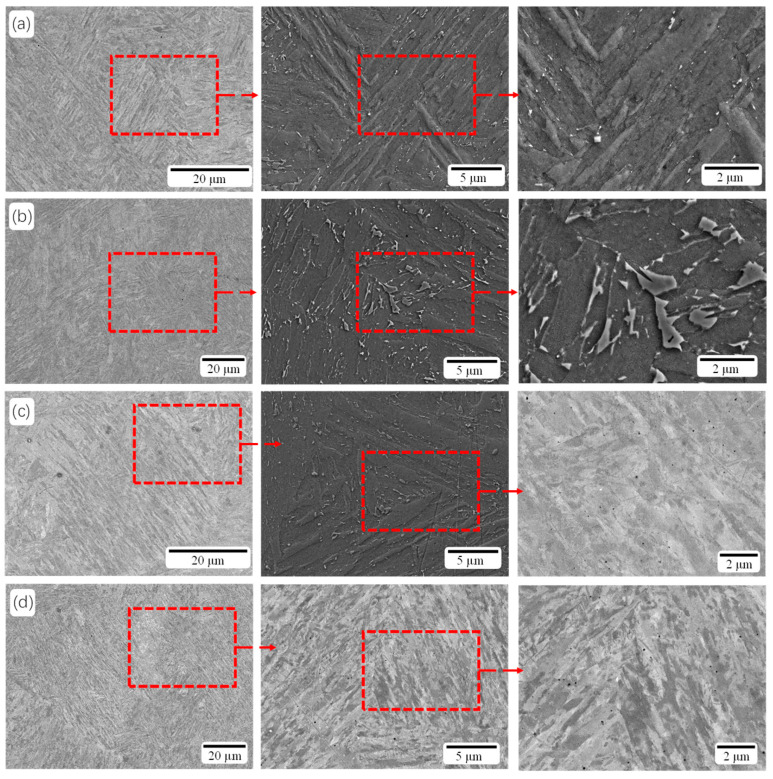
SEM images of the samples after SAT: (**a**) SAT 490-4, (**b**) SAT 490-7, (**c**) SAT 490-10, and (**d**) SAT 700-7.

Figure 5 shows the SEM images of the horizontal surface specimen after solution aging treatment (SAT). After SAT, the microstructure is fully homogenized, exhibiting fine, slate-like martensite with a small amount of austenite particles dispersed throughout. The original cellular and elongated structures are no longer present. Their disappearance is attributed to phase transitions during heat treatment and the precipitation of intermetallic compounds at grain boundaries. After aging treatment at 490 °C, an increase in austenite particles is observed with longer holding times (Figure 5a–c). Overall, the sample aged for 7 h shows the highest quantity of austenite particles. Slate-like martensite with white particles was also observed in the tissue of the SAT 700-7 sample.

### 3.2. Tensile Properties and Hardness

**Figure 6:**
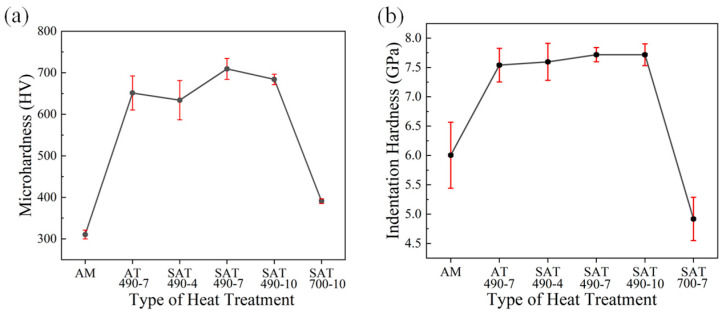
Hardness of horizonal surface of samples before and after heat treatment: (**a**) microhardness, (**b**) nanohardness.

Figure 6 shows the microhardness and nanohardness of the horizontal surface of samples before and after heat treatment. Compared to the untreated samples, the hardness of the specimens treated at 490 °C was significantly improved. The nanohardness of the SAT 490-7 sample was the highest, at approximately 7.7 GPa and 710 HV. However, the nanohardness of the SAT 700-10 sample was about 4.9 GPa, which is lower than that of the untreated samples, and the microhardness was also reduced. This decrease is attributed to differences in grain size and grain boundary clarity, the presence of a large number of precipitates in the heat-treated material, increased grain size, and the blurring of grain boundaries.

**Figure 7:**
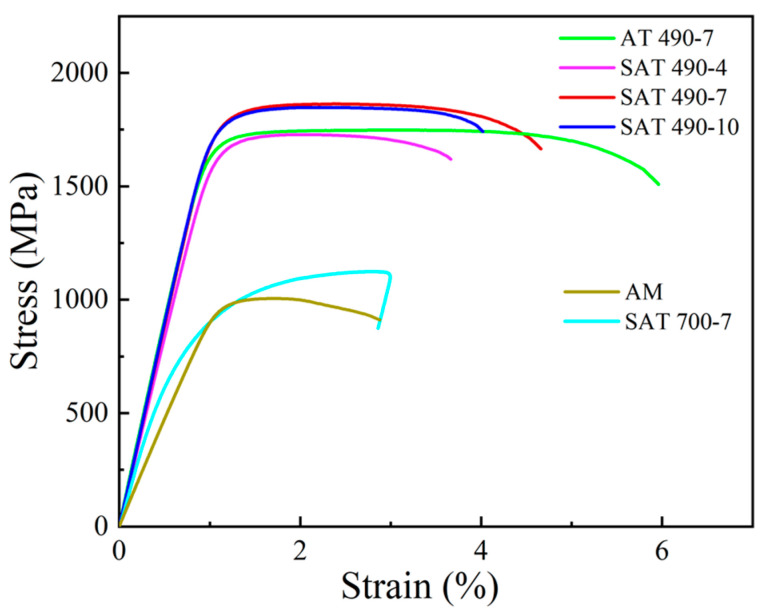
Tensile properties of the samples with different heat treatments.

The tensile properties (ultimate tensile strength, UTS, and percent elongation at fracture, El) of the heat-treated samples are summarized in Table 3 and Figure 7. The AT and SAT samples exhibit higher tensile strength and elongation compared to the AM samples. SAT 490-7 has the highest tensile strength at 1883 MPa; although, its elongation has decreased, with SAT 490-4 showing the lowest elongation at 3.5%. Overall, the SAT 490-7 sample demonstrates superior comprehensive mechanical properties.

**Figure 8:**
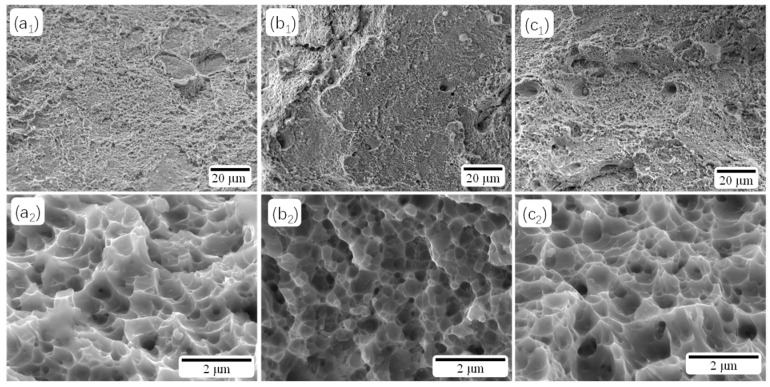
Fracture morphologies of the samples with different heat treatments: (**a_1_**,**a_2_**) AM, (**b_1_**,**b_2_**) AT 490-7, and (**c_1_**,**c_2_**) SAT 490-7.

Figure 8 shows examples of the fracture surfaces of AM, AT 490-7, and SAT 490-7 specimens. The fracture morphology of the AM specimens displays numerous cleavage planes and small dimples, indicative of brittle fractures. After aging treatment, the fracture dimples become shallower (Figure 8b_1_,b_2_), and the cross-section shows some undulations. After solution aging, the dimples become larger and deeper again (Figure 8c_1_,c_2_), with noticeable fluctuations in the cross-section, similar to the fracture morphology of the AM specimens. Overall, the fractures are relatively flat, resulting in lower elongation.

### 3.3. XRD Analyses

**Figure 9:**
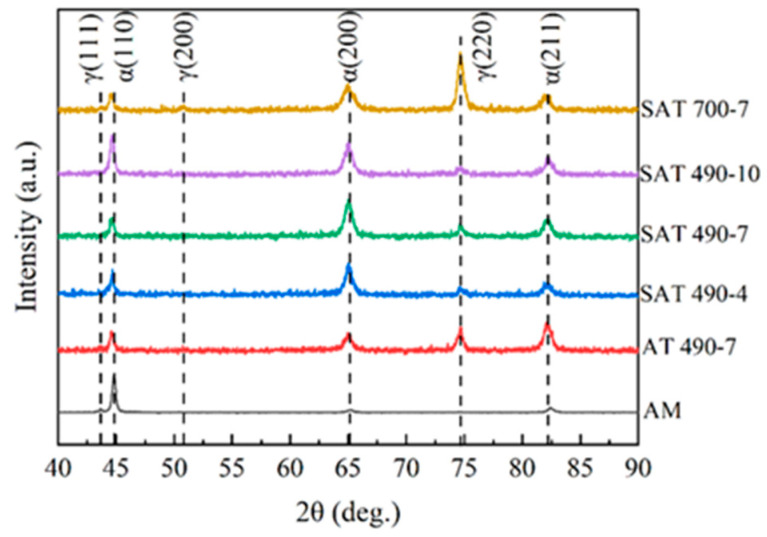
XRD analysis of the samples after different heat treatments.

The XRD patterns of the samples are presented in Figure 9. The AM specimens primarily consist of α-martensite with a small amount of γ-austenite phase. After aging treatment, the intensity of the γ (220) peak is significantly enhanced. Among the samples aged at 490 °C after solution treatment, the peak intensity of γ (220) is highest for SAT 490-7; although, it is still lower compared to the samples that underwent aging only.

Table 4 shows the weight percent of martensite and austenite in the samples before and after heat treatment. The Fe-Ni binary metastable phase diagram reveals the phase transition behavior. During the conversion of austenite to martensite, a particularly high cooling rate is not necessary; complete martensite can be achieved at a lower cooling rate. It has been observed [20] that the formation of reversed austenite (γ′) is nearly inevitable during the aging process. Based on this observation, it can be speculated that the increase in γ content in the AT specimens is likely due to the extended aging time, which gradually converts martensite into austenite, subsequently forming the γ′ phase. This transition process not only alters the phase composition of the specimen but can also significantly impact its performance. Therefore, it is crucial to strictly control the aging time to minimize the formation of reversed austenite and ensure the material’s performance and stability.

In martensitic aging steels, elements such as Mo, Ti, and Al form intermetallic compounds with Ni (Ni_3_Al, Ni_3_Ti, Ni_3_Mo), and Fe forms metal compounds (Fe_2_Mo) with Mo, which precipitate from the matrix during aging [2,4]. The addition of Co mainly serves to reduce the solubility of the Mo-containing strengthening phases and promotes the precipitation of additional strengthening phases during aging, thereby enhancing the strengthening effect [21]. Additionally, Mo can reduce the tempering brittleness of steel. In summary, it can be inferred that the structure of the martensitic steel (MS) after solution aging heat treatment consists of martensite, reversed austenite, and strengthening phases. The specific process is as follows:

$$\alpha +\gamma \overset{840\;{}^{\circ}C\;solution+quench+490\;{}^{\circ}C\;aging}{\to }\alpha +\gamma^{\prime }+{Ni}_{3}(Ti,Al,Mo)$$

### 3.4. TEM Analyses

**Figure 10:**
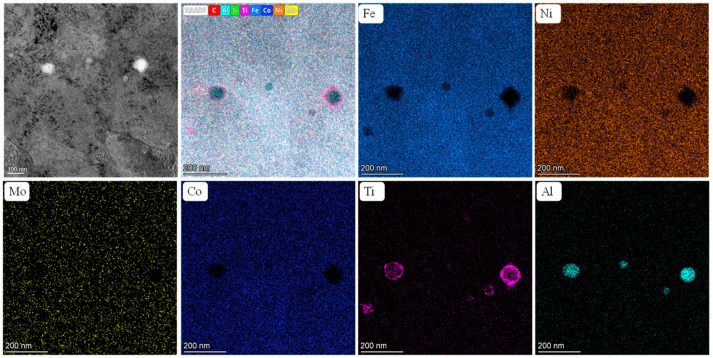
TEM images and EDX maps of the SAT 490-7 sample.

Figure 10 presents the TEM microstructure and EDX mapping of the SAT 490-7 sample. From the figure, it is evident that the sample exhibits the precipitation of spherical nanoparticles after aging, with diameters ranging from approximately 35 to 85 nm. The EDX map indicates that these nanoparticles primarily consist of Ti and Al. Due to their higher solid solubility and relatively fast diffusion rate in the solid solution, Ti and Al elements tend to initiate precipitation earlier than other elements. Once the Ti and Al elements reach their saturation solubility and exceed the solid solubility limit, they commence the formation of nano-sized precipitated phases. These precipitates effectively contribute to strengthening the alloy and enhancing its mechanical properties.

Conversely, Mo elements may possess a lower solid solubility or a higher diffusion rate, leading to their delayed saturation solubility compared to Ti and Al, and consequently, their precipitation occurs later. Numerous studies have established [22] that Ni_3_Ti is a primary strengthening phase in martensitic aging steels containing titanium, significantly impacting the alloy’s mechanical properties. Furthermore, Ti atoms can be partially substituted by Mo and Al. In conclusion, the aged sample features a strengthening phase formed through the reaction of Ni with Ti or Al.

### 3.5. EBSD Analyses

**Figure 11:**
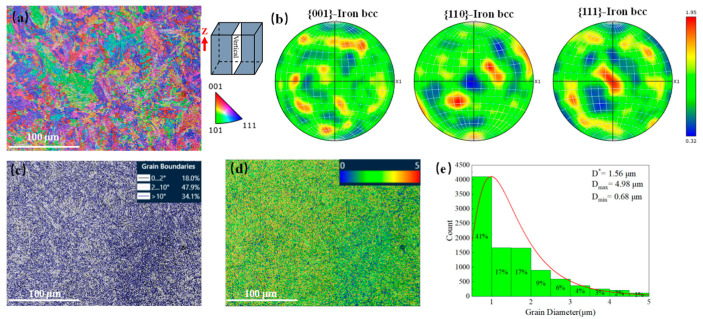
EBSD analysis of the AM sample: (**a**) IPF, (**b**) PFs, (**c**) GBs, (**d**) KAM, and (**e**) GSD.

Figure 11 shows the longitudinal section of the original additive-manufactured (AM) MS sample used for EBSD analysis. Figure 11a displays a color inverse pole figure (IPF), using [100], [010], [001], and [001] of the crystal coordinate system as the reference. The polar density projection in the Z-axis direction is shown, indicating that the grain orientation is random. Figure 11d presents the pole figure (PF), using the original coordinate system as the reference and the XY plane as the projection plane. The poles of each grain plane (001), (110), and (111) are plotted on the reference sphere, and the pole density distribution is projected at the equator. The color distribution is chaotic, and the maximum pole density value is less than 1.95, suggesting that the grain growth does not preferentially align along a specific crystal direction, and the texture is very weak.

Generally, grain growth occurs perpendicular to the melt pool boundary and opposite to the direction of heat conduction. This is because, during solidification, grain growth requires atoms or molecules to aggregate and crystallize in a liquid state to form a crystal nucleus, and this process is driven by the temperature gradient at the melt pool boundary, which is the highest. For cubic crystal materials, the <100> direction corresponds to the lowest crystal plane energy, making it more favorable for rapid crystal growth. Therefore, the <100> crystal direction is typically the preferred growth direction. However, the SLM-prepared MS samples do not exhibit a distinct Z-oriented texture, which may be attributed to the SLM interlayer scanning strategy. In SLM, an alternating interlayer rotation scanning strategy is often used to promote uniform heat distribution and reduce stress concentrations. This rotational scanning strategy results in alternating shifts in the direction of heat flow in each layer, which impedes the formation of grain textures along the Z direction [23,24].

Figure 11c shows the distribution of grain boundaries (GBs), with 65.9% comprising low-angle grain boundaries (LAGBs) and 34.1% high-angle grain boundaries (HAGBs) among the small-angle grain boundaries. Additionally, 18.0% of the subgrain boundaries (with a 2° difference between adjacent grain positions) are included in the small-angle grain boundaries. Generally, HAGBs have a high binding energy and contribute significantly to the grain boundary energy, forming primarily at the boundaries of martensitic structures. In contrast, LAGBs have lower binding energy and grain boundary energy, forming within the crystal. Dislocations in LAGBs can be easily arranged along grain boundaries, leading to the formation of arranged dislocations. Figure 11d shows the kernel average misorientation (KAM) diagram, indicating that the residual microscopic stress on the surface of the specimen after SLM molding is minimal. Figure 11e presents the grain size distribution (GSD), with a maximum grain diameter of 4.98 μm, a minimum diameter of 0.68 μm, and an average diameter of 1.56 μm.

**Figure 12:**
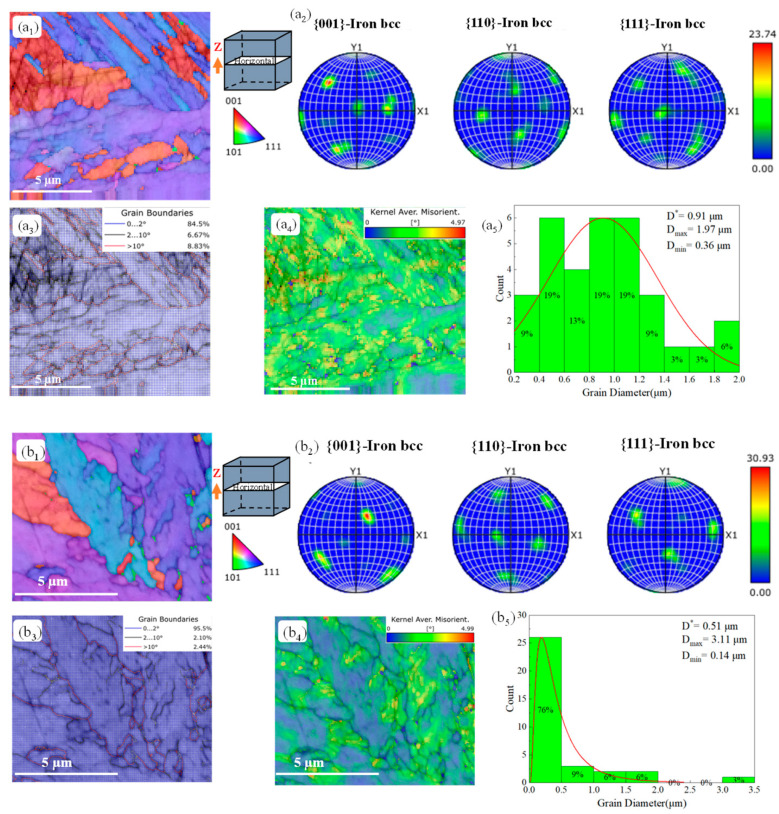
EBSD analysis of the samples after heat treatments: (**a_1_**–**a_5_**) SAT 490-7 and (**b_1_**–**b_5_**) AT 490-7.

Figure 12 shows the EBSD analysis of a cross-section of a sample after heat treatment. The reverse pole figure diagrams (Figure 12a_1_,b_1_) and pole figure diagrams (Figure 12a_2_,b_2_) for each sample reveal a clear <100> texture preference in the heat-treated sample. The grain boundary distribution plots (Figure 12a_3_,b_3_) show a significant reduction in high-angle grain boundaries after heat treatment, with all being below 10%. The average dislocation density plots (Figure 12a_4_,b_4_) indicate that residual stresses are minimal and are primarily concentrated near the high-angle grain boundaries. Additionally, the particle size distribution plots (Figure 12a_5_,b_5_) demonstrate a decrease in the equivalent circle diameter of the grains following heat treatment. This reduction may be attributed to the dissolution of solute atoms into the grain interiors during the solution treatment phase, which alleviates local supersaturation near the grain boundaries and promotes the dissolution of precipitates at the large-angle grain boundaries, thereby reducing their number. Both solution treatment and aging treatment facilitate grain recrystallization and regrowth. This process leads to a decrease in grain size, as larger grains are replaced by smaller regrowing grains. Simultaneously, grain boundary migration may occur, with smaller grains potentially consuming adjacent larger grains, resulting in a reduction in the overall grain size.

## 4. Conclusions

In this work, the effects of heat treatments on microstructures and properties of a martensitic aging steel 18Ni300 prepared by SLM were investigated. The following conclusions are drawn:

(1) Heat treatment significantly alters the micromorphology of the original sample. The cross-sectional topography of the as-built specimen is characterized by long strips of laser tracks, while the longitudinal cross-sectional topography exhibits a fish scale-like molten pool pattern. After heat treatment, these topographical features are no longer visible. The microstructure of the original and aged samples reveals a cellular structure and long strip structure. Following solution aging heat treatment, both the cellular and long strip structures are completely eliminated, resulting in a bundle-like arrangement that is nearly parallel and of almost uniform length. Additionally, inverted austenite can be observed. For samples aged at 700 °C, white particles of Mo are present.
(2) TEM and XRD results indicate that the structure of the martensitic steel transitions from a martensitic phase and austenite phase to a martensitic phase, inverted austenite phase, and Ni_3_(Ti, Al, Mo) precipitated phase after solution aging heat treatment.
(3) Heat treatment improves the hardness and tensile strength of the sample. Prior to heat treatment, the specimen had a hardness of 310 HV, a tensile strength of 1002 MPa, and an elongation to fracture of 2.9%. After heat treatment, the samples subjected to solution treatment at 820 °C for 2 h followed by aging at 490 °C for 7 h exhibited the best overall properties, with a microhardness of 710 HV, a nanohardness of 7.7 GPa, a tensile strength of 2068 MPa, and an elongation at break of 4.5%.

This study successfully fabricated high-performance 18Ni-300 maraging steel and conducted a preliminary exploration of the effects of different heat treatment processes on its microstructure and properties. However, due to time and experimental constraints, there is still room for further investigation. During service, 18Ni300 maraging steel is often exposed to high-temperature molten aluminum, and its corrosion resistance in such environments is a key indicator of performance. Therefore, future research could focus on designing specialized molten aluminum corrosion tests to study the material’s corrosion resistance in these conditions.

## Figures and Tables

**Table 1:** Chemical composition of 18Ni300 maraging steel powder (wt%).

Ni	Co	Mo	Ti	Al	Fe
17.5–18.5	8.50–10.0	4.50–5.00	0.80–1.10	0.05–0.15	Bal
C	Si	Mn	P	S	Cr
≤0.03	≤0.10	≤0.15	≤0.010	≤0.010	≤0.60

**Table 2:** Heat treatments used in this study.

	Heat Treatments
/	No heat treatment after fabrication (AM)
Aging	490 °C-7h (AT 490-7)
Solution+ Aging	820 °C-2h-Water quench + 490 °C-4 h/7h/10 h, Air cooling (SAT 490-4/SAT 490-7/SAT 490-10)
	820 °C-2h-Water quench + 700 °C-7 h, Air cooling (SAT 700-7)

**Table 3:** Mechanical properties of the 18Ni300 samples.

Specimens	UTS (MPa)	EL (%)
AM	1002	2.9
AT 490-7	1782	5.7
SAT 490-4	1826	3.5
SAT 490-7	1883	4.5
SAT 490-10	1864	4.2
SAT 700-7	1135	6.1

**Table 4:** Martensite and austenite fractions of the samples after heat treatments.

Specimens	α (wt.%)	γ (wt.%)
AM	93.4	6.6
AT 490-7	48.2	51.8
SAT 490-4	82.2	17.8
SAT 490-7	73.8	19.5
SAT 490-10	84.2	15.7
SAT 700-7	26.7	73.3

## Data Availability

The original contributions presented in this study are included in the article. Further inquiries can be directed to the corresponding authors.
